# Frequency of COVID‐19 vaccine side effects and its associated factors among the vaccinated population of Pakistan: A cross‐sectional study

**DOI:** 10.1002/hsr2.1071

**Published:** 2023-01-18

**Authors:** Farah Yasmin, Hala Najeeb, Hasan Fareed Siddiqui, Muhammad Sohaib Asghar, Hashir Ali Awan, Rana Muhammad Usama, Zoha Allahuddin, Muhammad Junaid Tahir, Kaleem Ullah, Mohammed Mahmmoud Fadelallah Eljack

**Affiliations:** ^1^ Department of Medicine, Dow Medical College Dow University of Health Sciences Karachi Pakistan; ^2^ Department of Medicine, Dow University Hospital‐Ojha Campus Dow University of Health Sciences Karachi Pakistan; ^3^ Department of Medicine Lahore General Hospital Lahore Pakistan; ^4^ Department of Radiology Pakistan Kidney and Liver Institute and Research Center (PKLI & RC) Lahore Pakistan; ^5^ Department of Surgery, Liver Transplant and Hepatobiliary Unit Pir Abdul Qadir Shah Jelani Institute of Medical Sciences Gambat Pakistan; ^6^ Department of Community Medicine, Faculty of Medicine and Health Sciences University of Bakht Alruda Ad Duwaym Sudan

**Keywords:** adverse effects, COVID‐19 vaccine, postvaccination symptoms, SARS‐CoV‐2 vaccine, vaccine side effects

## Abstract

**Background:**

Coronavirus disease 2019 (COVID‐19) vaccine side effects have an important role in the hesitancy of the general population toward vaccine administration. Therefore, this study was conducted to document the COVID‐19 vaccine side effects in our population.

**Materials and Methods:**

An online survey‐based, cross‐sectional study was carried out from September 1, 2021, to October 1, 2021, to document the side effects of the COVID‐19 vaccine among the general public. The questionnaire included participants’ sociodemographic data, type of vaccine, comorbidities, previous COVID‐19 infection, and assessment of side effects reported by them.

**Results:**

The majority of the participants were <20 years of age (62.2%), females (74.9%), belonged to the educational sector (58.1%), residents of Sindh (65.7%), and were previously unaffected by COVID‐19 infection (73.3%). Sinovac (38.7%) followed by Sinopharm (30.4%) and Moderna (18.4%) were administered more frequently. Commonly reported side effects were injection site pain (82%), myalgia (55%), headache (46%), fatigue/malaise (45%), and fever (41%). Vaccine side effects were more likely to be reported with the first dose as compared to the second dose. On regression analysis, factors associated with occurrence of side effects included younger age (odds ratio [OR]: 6.000 [2.065–17.431], *p* < 0.001), female gender (OR: 2.373 [1.146–4.914], *p* = 0.020), marital status (OR: 0.217 [0.085–0.556], *p* < 0.001), graduate level of education (OR: 0.353 [0.153–0.816], *p* = 0.015), and occupation being either retired, freelancers, or social workers (OR: 0.310 [0.106–0.909]), *p* = 0.033). Previous infection with COVID‐19 (*p* = 0.458) and comorbidities were found unrelated (*p* = 0.707) to the occurrence of side effects.

**Conclusion:**

The overall prevalence of local side effects was quite higher than the systemic ones. Further large‐scale studies on vaccine safety are required to strengthen public confidence in the vaccination drive.

## 1 INTRODUCTION

With over 250 million cases,^1^ the coronavirus disease 2019 (COVID‐19) pandemic had virtually brought the entire world to a standstill and created a massive public health emergency. The global vaccination drive against the deadly viral disease has provided a sliver of hope for a return to normalcy, and since early 2021, more than 7.5 billion vaccine doses have been administered worldwide.^1^

The World Health Organization (WHO) approved several vaccines for use, and many are under trial.^2^, ^3^ Messenger RNA (mRNA) vaccines such as Pfizer‐BioNTech (BNT162b2) and Moderna (mRNA‐1273), inactivated vaccines such as Sinopharm and Sinovac, and Adenovirus vector‐based vaccines such as Sputnik V (Gamaleya) and AstraZeneca (AZD1222 Vaxzevria) are among those that the WHO has finalized and readily available across the world via the COVAX vaccine‐distribution program.^2^, ^4^ In addition to the vaccines mentioned, the single‐dose viral‐vector‐based vaccine CanSinoBIO (Ad5‐nCoV) (also called PakVac due to local production) is available to the public above the age of 12 in Pakistan.^5^ According to the National Command and Operation Center (NCOC), more than 120 million vaccine doses have been administered in Pakistan.^5^

Despite approvals by concerned authorities and the apparent protection provided by vaccines, fears regarding their safety are still a concern. Despite their availability, inoculation's short‐ and long‐term side effects have increased vaccine hesitancy in many parts of the world. Large‐scale surveys and meta‐analyses had predicted widespread vaccine hesitancy due to concerns regarding a potential COVID‐19 vaccine's safety and possible side effects.^6^, ^7^, ^8^ A study across seven European countries showed that nearly 55% of the participants who showed vaccine hesitancy cited fear of side effects as their main reason.^9^ Parents were hesitant to allow COVID‐19 vaccination for their children due to anxiety associated with vaccine side effects.^10^ Other studies have reported that the general population will be more willing to get vaccinated if the fear of side effects is addressed.^11^ A study in China also affirmed that the side effects remain a significant cause of concern for the general population, primarily the inactivated vaccines.^12^ More recently, surveys targeting various sociodemographic cohorts have shown persistent vaccine hesitancy. Although the ratio is declining, nearly 10% of the population was hesitant about or not getting the vaccine in Thailand.^13^ In Saudi Arabia, a cross‐sectional study confirmed that fear of side effects was the major driving factor behind vaccine hesitancy.^14^

On the other hand, 31.5% of parents in Italy were hesitant in vaccinating their children,^15^ and 25% of young adults also expressed their hesitance in receiving a COVID‐19 vaccine.^16^ The fast‐paced approval process allowed reasonable doubt to be cast upon whether an adequate time for safety trials and assessment was allocated to these vaccines before being made public. The influence of political agendas on such trials was associated with the lower willingness of the general public to voluntarily inoculate themselves with a COVID‐19 vaccine.^9^, ^11^, ^17^

Before the COVID‐19 pandemic, WHO had already included vaccine hesitancy among the Top 10 threats to public health in 2019.^18^ Pakistan has a well‐established precedent of widespread hesitancy toward polio vaccines.^19^ Due to this, the acceptability of COVID‐19 vaccines in Pakistan is of utmost importance and possibly linked with its potential side effects. Therefore, this study aims to collect and document empirical data for vaccine side effects in the general population of Pakistan.

## 2 METHODS

### 2.1 Study design, participants, and protocol

An online survey‐based, cross‐sectional study was carried out from September 1, 2021, to October 1, 2021, to estimate the prevalence of COVID‐19 vaccine side effects among the general population of Pakistan. A self‐administered online survey was circulated via social media platforms (i.e., Facebook, WhatsApp, Instagram, and Twitter).

After an extensive literature search on PubMed/Medline, Embase, and Google Scholar, the questionnaire enlisted commonly reported side effects. While designing the study, Strengthening the Reporting of Observational Studies in Epidemiology (STROBE) guidelines for cross‐sectional studies were followed.^20^ The questionnaire consisted of four sections:

‐ Sociodemographic details of participants, including gender, age, marital status, education level, occupation, monthly household income, and the region of residence.
‐ Vaccine‐related data: the COVID‐19 vaccine received and the date of dosages.
‐ Medical histories such as comorbidities, previous COVID‐19 infection, and the time between vaccination and infection.
‐ The side effects of the COVID‐19 vaccine after the first or the second dose, severity, and duration (<1, 1–6, 6–24, >24 h), respectively.

The listed side effects were obtained from existing studies^21^, ^22^, ^23^ and consulting public health specialists. These were divided into general (myalgia, malaise, fever, headache, chills, arthralgia, nausea, vomiting, abdominal pain, chest tightness); oral (xerostomia, swollen lips, taste disturbances, tongue‐tingling, cheilitis, bleeding gingiva, ulcers, halitosis), and dermatological side‐effects (injection site pain, swelling or redness, lymphadenopathy, blisters).

The study did not collect the personal information of participants and anonymity was maintained. The ethical approval was obtained by the Institutional Review Board of PAQSJ Institute of Medical Sciences, Gambat, Pakistan (Reference Number: ERC/21/9). An introduction to the study was given in the questionnaire. The participants were informed of the purpose of the study, and informed consent was obtained. Participants could withdraw from the study by reaching out to the research team.

### 2.2 Sample size and statistical analysis

The sample size was calculated using Open‐EPI, considering a response rate of 80%, with a 5% margin of error and a 95% confidence interval. Statistical analysis was performed using SPSS version 25.0 (SPSS Inc.). Descriptive statistics were performed to obtain the frequencies for side‐effects, severity, and duration of side‐effects by dosage interval of vaccines, and the occurrence of side‐effects with dosage intervals. A multiple regression analysis was performed to analyze the demographic characteristics (age, sex, marital status, employment status, occupation, previous COVID‐19 infection, comorbidities) that affected the occurrence of post‐COVID‐19 vaccine complications. A *p* Value < 0.05 was considered statistically significant for all outcomes.

## 3 RESULTS

A total of 434 responses were considered valid and contributed to the results. Two hundred seventy (62.2%) participants were under the age of 20, 141 (32.5%) participants were aged 21–30, and 23 (5.3%) participants were above the age of 30. The sample consisted of 109 male (25.1%) and 325 female patients (74.9%). Four hundred two (92.6%) were single, while 3 (0.7%) were divorced or widowed, and 29 (6.7%) participants were married. Sixty (13.8%) had a university degree, those with secondary school were 116 (26.7%), and those currently enrolled in an undergraduate program were 258 (59.4%). By occupation, most participants belonged to the education sector (*n* = 252, 58.1%), followed by freelancer or social workers (*n* = 55, 12.7%), healthcare workers (*n* = 17, 3.9%), enterprise workers (*n* = 17, 3.9%), and 93 (21.4%) of the participants were currently unemployed. Among different areas of Pakistan, a majority belonged to the province of Sindh (*n* = 285, 65.7%), followed by Punjab (*n* = 135, 31.1%), Khyber Pakhtunkhwa (*n* = 12, 2.8%), and Balochistan (*n* = 2, 0.5%) as shown in Table 1.

**Table 1:** Baseline and demographic characteristics of the study population (*n* = 434)

Variables	Characteristics	Frequency	Percentage
Age (in years)	<20	270	62.2
	21–30	141	32.5
	30 above	23	5.3
Gender	Male	109	25.1
	Female	325	74.9
Marital status	Single	402	92.6
	Married	29	6.7
	Divorced/widowed	3	0.7
Education level	Graduate	60	13.8
	Secondary/higher‐secondary	116	26.7
	Undergraduate	258	59.4
Monthly household income (PKR)	<20,000	75	17.3
	<50,000	69	15.9
	<100,000	87	20.0
	100,00–200,000	101	23.3
	>200,000	102	23.5
Occupation	Enterprise or institution workers	17	3.9
	Healthcare worker	17	3.9
	Others (freelancers, retirees, and social workers)	55	12.7
	Teachers/students	252	58.1
	Unemployed	93	21.4
Region	Sindh	285	65.7
	Punjab	135	31.1
	Khyber Pakhtunkhwa	12	2.8
	Balochistan	2	0.5
Previously infected with COVID‐19	Yes	106	24.4
	No	318	73.3
	Infected after vaccine	10	2.3
Type of vaccine administered	Astrazeneca	10	2.3
	Cansino	19	4.4
	Johnson & Johnson	1	0.2
	Moderna	80	18.4
	Pakvac	5	1.2
	Pfizer	17	3.9
	Sinopharm	132	30.4
	Sinovac	168	38.7
	Sputnik	1	0.2
	Sputnik V	1	0.2
Comorbidities	None	344	79.3
	Yes	90	20.7

*Note*: Data are presented as frequency and percentages.

Nearly three‐quarters of the participants (*n* = 318, 73.3%) were not infected with COVID‐19 previously, while a quarter (*n* = 106, 24.4%) had a history of COVID‐19. Ten participants (2.3%) were infected after they received a vaccine. Among the types of vaccines received by participants, a majority received Sinovac (*n* = 168, 38.7%) and Sinopharm (*n* = 132, 30.4%). Sputnik (or Sputnik V) was received by two participants (0.4%). mRNA vaccines such as Pfizer BioNTech (*n* = 17, 3.9%) and Moderna (*n* = 80, 18.4%) were received by a total of 22.3% participants. Other vaccines included AstraZeneca (*n* = 10, 2.3%), CanSinoBIO (*n* = 19, 4.4%), and Johnson & Johnson (*n* = 1. 0.2%). 79.3% of the participants (*n* = 344) reported no significant comorbidities, while 20.7% (*n* = 90) reported having one or more comorbidities.

**Figure 1:**
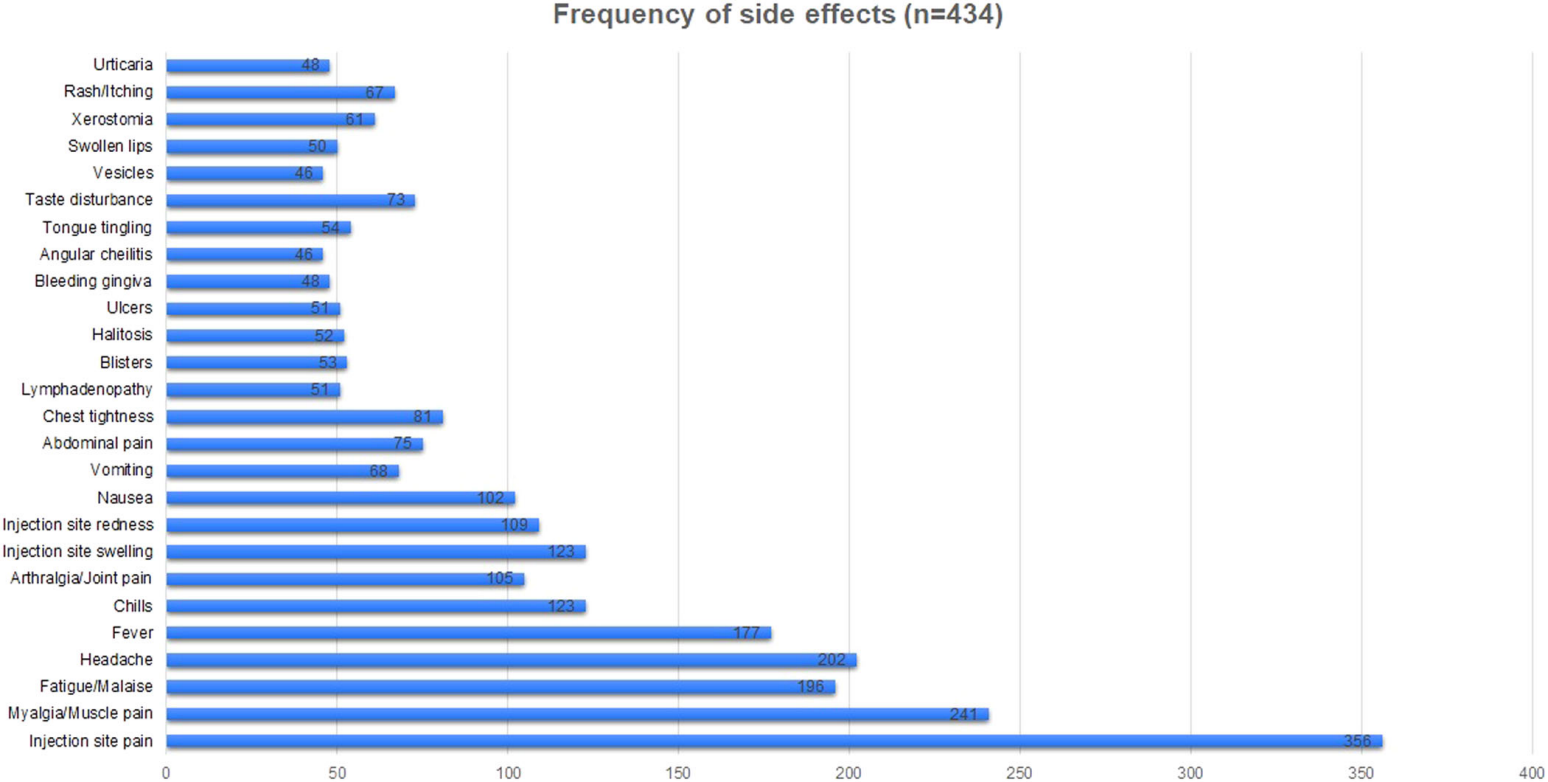
Frequency of side effects reported by study population (*n* = 434)

**Figure 2:**
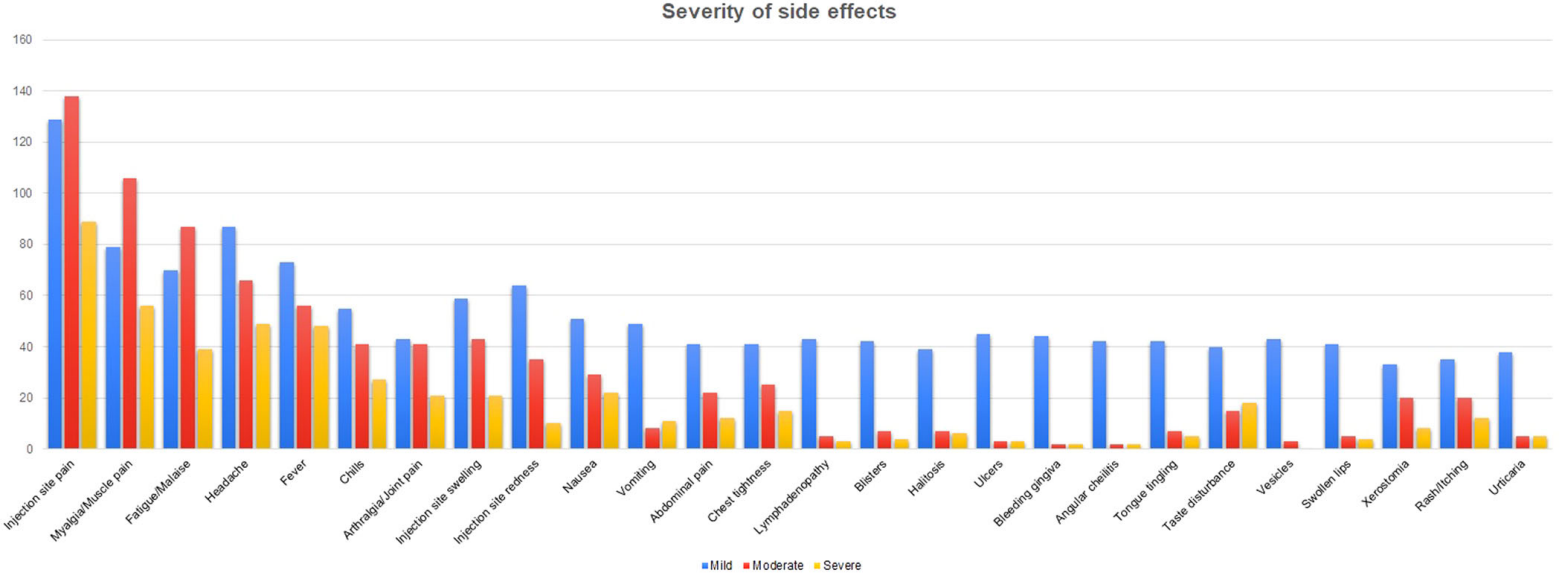
Reported severity of side effects experienced on a scale of mild, moderate to severe by the study participants

**Figure 3:**
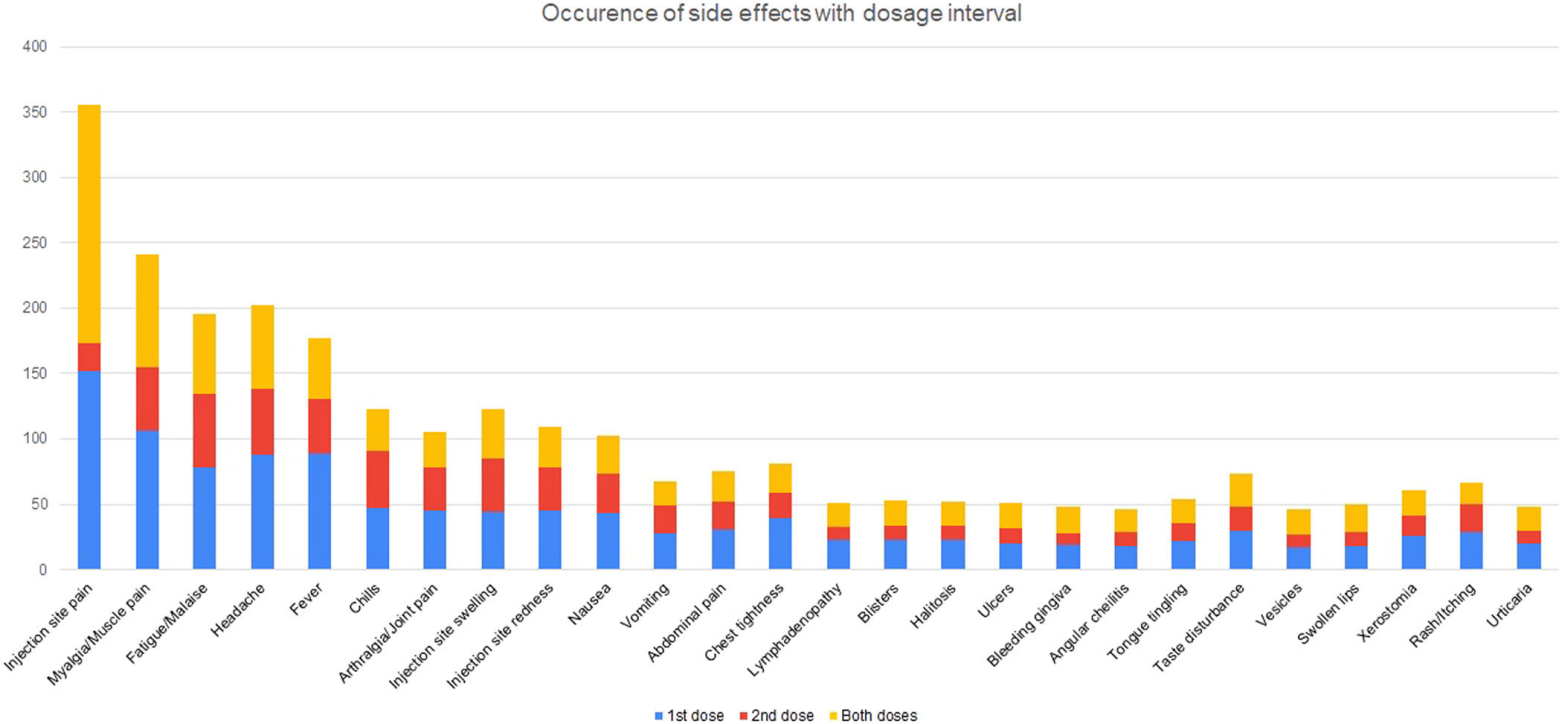
Occurrence of side effects with dosage interval of coronavirus disease 2019 vaccine

The most common side‐effect reported by participants was pain at the injection site (*n* = 356, 82.0%), as shown in Figure 1. Other common side‐effects were myalgia (*n* = 241, 55.5%), headache (*n* = 202, 46.5%), fatigue or malaise (*n* = 196, 45.1%), and fever (*n* = 177, 40.8%). The least common side‐effects were angular cheilitis (*n* = 46, 10.6%), the appearance of vesicles (*n* = 46, 10.6%), bleeding gingiva (*n* = 48, 11.1%), urticaria (*n* = 48, 11.1%), and swollen lips (*n* = 50, 11.5%). While most side‐effects were mostly mild, pain at the injection site, myalgia, and fatigue or malaise were moderate in severity, as shown in Figure 2. Vaccine side effects were more common upon administration of the first dose than the second dose, as shown in Figure 3.

**Figure 4:**
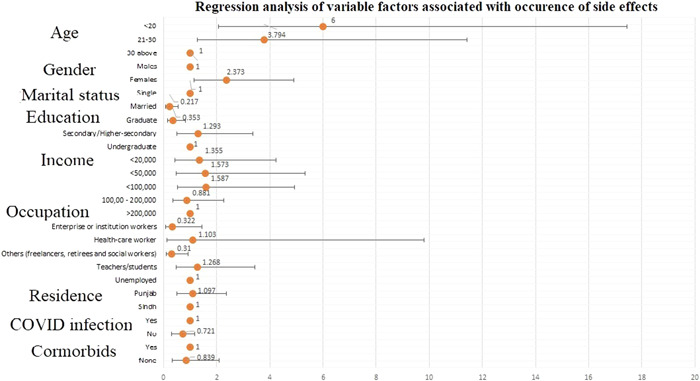
Multivariate analysis of factors associated with occurrence of side effects

**Figure 5:**
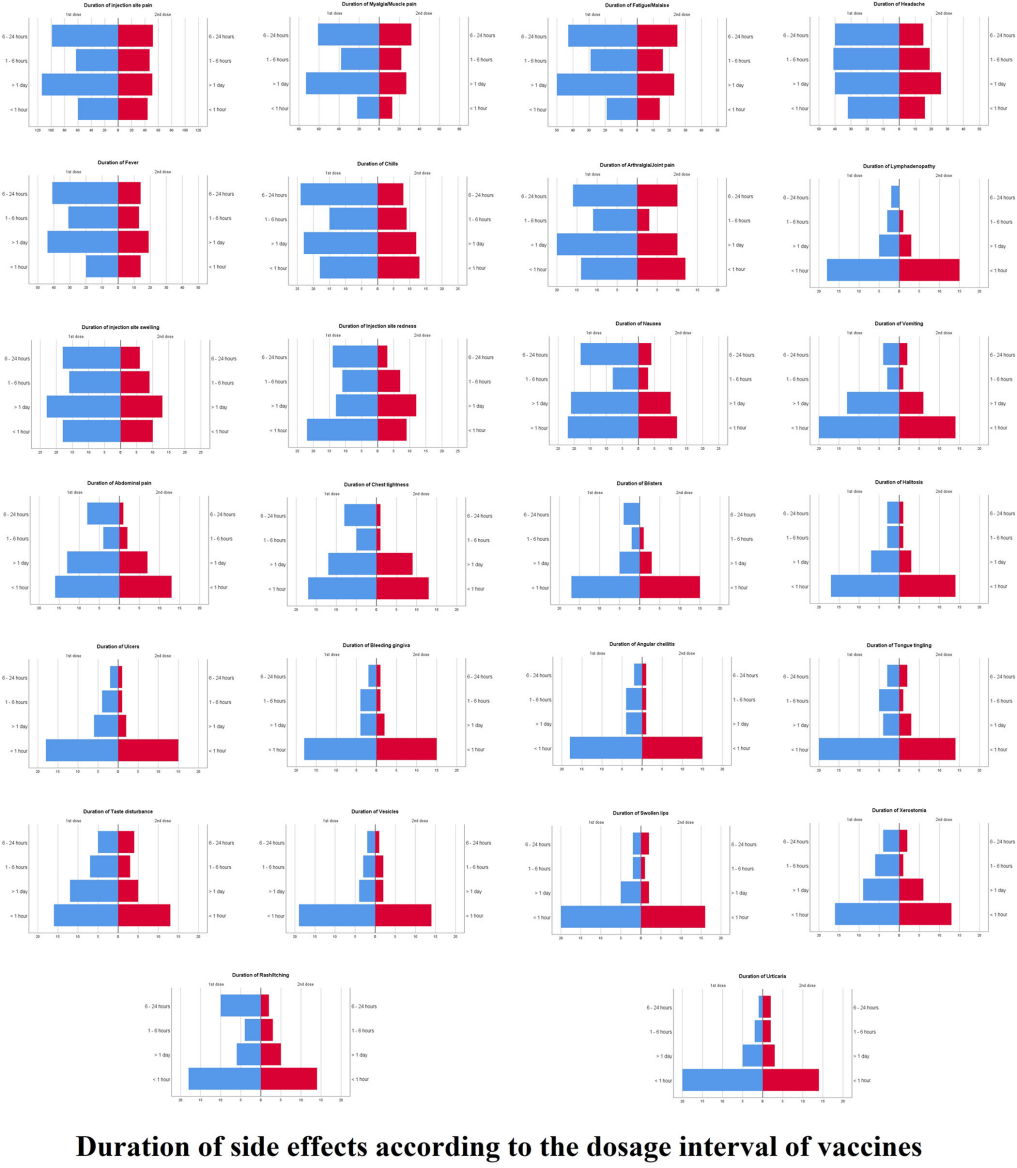
Duration of side effects according to dosage interval of vaccine

Upon regression analysis, age below 20 (odds ratio [OR] = 6.000 [2.065–17.431], *p* < 0.001) or between 21 and 30 (OR = 3.794 [1.259–11.430], *p* = 0.018), being married (OR = 0.217 [0.085–0.556], *p* < 0.001), female gender (OR = 2.373 [1.146–4.914], *p* = 0.020), having a university degree (OR = 0.353 [0.153–0.816], *p* = 0.015), and employment as a freelance worker, social workers, or being a retiree (OR = 0.310 [0.106–0.909], *p* = 0.033), were significantly associated with having side‐effects of postvaccination as shown in Figure 4. The previous history of being infected with COVID‐19 (*p* = 0.458) and comorbidities (*p* = 0.707) was not associated with the appearance of side effects after COVID‐19 vaccination, as shown in Table 2. The duration of side effects is reported in Figure 5.

**Table 2:** Regression analysis of variable factors associated with the occurrence of side effects

Variables	Odds ratio (95% CI)	*p* Value
Age (in years)		
<20	6.000 (2.065–17.431)	0.001
21–30	3.794 (1.259–11.430)	0.018
30 above	Ref	‐
Gender		
Male	Ref	‐
Female	2.373 (1.146–4.914)	0.020
Marital status		
Single	Ref	‐
Married	0.217 (0.085–0.556)	0.001
Divorced/widowed	a	a
Education level		
Graduate	0.353 (0.153–0.816)	0.015
Secondary/higher‐secondary	1.293 (0.496–3.370)	0.599
Undergraduate	Ref	‐
Monthly household income (PKR)		
<20,000	1.355 (0.435–4.221)	0.600
<50,000	1.573 (0.464–5.325)	0.467
<100,000	1.587 (0.511–4.927)	0.424
100,00–200,000	0.881 (0.342–2.267)	0.792
>200,000	Ref	‐
Occupation		
Enterprise or institution workers	0.322 (0.072–1.437)	0.138
Healthcare worker	1.103 (0.124–9.792)	0.930
Others (freelancers, retirees, and social workers)	0.310 (0.106–0.909)	0.033
Teachers/students	1.268 (0.467–3.440)	0.641
Unemployed	Ref	‐
Region		
Sindh	Ref	‐
Punjab	1.097 (0.507–2.376)	0.814
Khyber Pakhtunkhwa	a	a
Balochistan	a	a
Previously infected with COVID‐19		
Yes	Ref	‐
No	0.721 (0.304–1.170)	0.458
Comorbidities		
None	0.839 (0.335–2.097)	0.707
Yes	Ref	‐

Abbreviations: CI, confidence interval; Dependent variable, experienced no side effects vs experienced at least one side effects; Ref, reference category.

^a^ Could not be computed because of low response distribution.

## 4 DISCUSSION

Coronavirus has continued to affect millions of people, causing the loss of lives of millions of people and catastrophic destructions to economies worldwide. Vaccination of the masses to create herd immunity is the most critical step to ending this pandemic, and multiple vaccines are being used globally to provide immunization to the public. Despite the worldwide approval of COVID‐19 vaccines and the evident protection, the safety of the vaccines and fear about their side effects has continued to plague the immunization drives. This cross‐sectional study aims to find the prevalence of side effects in the general population in Pakistan who underwent COVID‐19 vaccination.

There were multiple side effects reported by the patients, with the vaccine's adverse effects more prominent after the second dose than in the first dose. This finding can be interpreted based on the body's immune response, due to preciously triggered immune system by the first dose. The intense immune response to the second dose is possibly mediated by cytokines to cause an inflammatory response in muscles, blood vessels, and other tissues. The immune response could also cause flu‐like symptoms, even days after administering the vaccine.^24^

The most common side effects that were reported included pain at the injection site, myalgia (*n* = 241, 55.5%), headache (*n* = 202, 46.5%), fatigue or malaise (*n* = 196, 45.1%), and fever (*n* = 177, 40.8%). The most prominent among these was having pain at the injection site, present in 82% of the involved participants. This result was comparable to a similar study conducted in Saudi Arabia and by the study conducted by the Centers for Disease Control and Prevention (CDC); however, a study conducted in the United Kingdom by Cristina et al. showed much lower rates of pain at the injection site.^25^, ^26^, ^27^

Our data for fatigue and headache also showed a higher trend than the previous studies. A study by Riad et al.^28^ showed a lower frequency for both aforementioned side effects, with fatigue present in 23.6% and headache found in 18.7% of the included participants. This study's results also mirror the study conducted by the CDC in the aspect that compared to the pain, swelling, and redness, both were less frequently reported.^27^ With regard to the duration of these side effects, the current study reported that most of the side effects had longer durations for the first dose than the second.

The data gathered in our study showed an increased percentage of adverse effects after the administration of the second dose as compared to the first dose. This finding was also consistent with previously published data by Menni et al.,^26^ which also showed an increased incidence of adverse effects after administering the second dose. This could be due to an increase in immunogenicity due to vaccination, leading to an increase in reactogenicity.^26^ Comorbidities were found to have no significant impact on the incidence of side effects, according to Choi and Cheong.^29^ The duration of side effects, specifically injection site pain, injection site swelling, myalgia, fatigue, headache, fever, and chills, was markedly longer after the first dose of the vaccine compared to the second dose in our study.

Regression analysis also indicated that women were generally more likely to have adverse effects from the vaccine than men. This finding was similar to the other previously conducted studies by Jayadaven et al.,^30^ Riad et al.,^28^ and El‐Shitany et al.^25^, ^31^ This difference in side effects between genders is consistent with the side effects for other inactivated virus vaccines, such as influenza, attenuated dengue vaccines, and measles, mumps and rubella combination vaccines. This can be attributed to women reporting side effects more frequently and showing more robust immune responses.^31^

The younger population in our study, especially participants under the age of 30, showed a higher incidence of side effects of the vaccine. The reason may be a predominant immune response in young which dampens with aging. This finding aligned with the study results by Saeed et al.,^32^ which also showed younger adults being more prone to the side effects of immunization. The reactogenicity of the vaccines is known to be interrelated with the transient elevation of inflammatory cytokines, suggesting that the reactiveness declines with age.^33^ However, this should not be a dependable sign for an appropriate immune response. The strength of this study includes the fact that it consisted of participants from across the country, giving a good overall indication of the adverse effects of the vaccine in the country.

This study had a few limitations. A small sample size presents a major limitation of our cross‐sectional analysis. Moreover, the participants were asked to recall past adverse effects, which could have led to recall bias. Apart from this, participants of this study included only those who had access to the Internet, leading to sampling bias. Future studies should counter these limitations by collecting data via a community‐based approach to gather data from all age groups, gender, and socioeconomic segments of society. Our study also does not specify side effects based on individual vaccines, and future studies should focus on addressing this notion. Furthermore, due to the usage of self‐reported data, there could be a presence of reporting bias in our study. This article did not analyze long‐term side‐effects, which need to be looked upon further in future studies.

This publication would help enhance the vaccination rates in our country by expelling myths that have continued to hamper the vaccination drives and making the population more confident in getting inoculated by knowing what to expect postvaccination.

## 5 CONCLUSION

The overall frequency of local side effects was relatively higher than the systemic ones. Further large‐scale studies on vaccine safety are required to strengthen public confidence in the vaccination drive. This study would help enhance the vaccination rates in our country by expelling myths that have continued to hamper the vaccination drives and making the population more confident in getting inoculated by knowing what to expect postvaccination.

## AUTHOR CONTRIBUTIONS

**Farah Yasmin**: conceptualization; writing – original draft. **Hala Najeeb**: data curation; formal analysis; writing – original draft. **Hasan Fareed Siddiqui**: data curation; writing – original draft. **Muhammad Sohaib Asghar**: conceptualization; data curation; formal analysis. **Hashir Ali Awan**: data curation; writing – review and editing. **Rana Muhammad Usama**: writing – review and editing. **Muhammad Junaid Tahir**: Writing – review and editing. **Kaleem Ullah**: writing – review and editing. **Mohammed Mahmmoud Fadelallah Eljack**: writing – review and editing. All authors have read and approved the final version of the manuscript, corresponding author had full access to all of the data in this study and takes complete responsibility for the integrity of the data and the accuracy of the data analysis.

## CONFLICT OF INTEREST

The authors declare no conflict of interest.

## TRANSPARENCY STATEMENT

The lead author Mohammed Mahmmoud Fadelallah Eljack affirms that this manuscript is an honest, accurate, and transparent account of the study being reported; that no important aspects of the study have been omitted; and that any discrepancies from the study as planned (and, if relevant, registered) have been explained.

## Data Availability

the data that support the findings of this study are available from the corresponding author upon reasonable request.

## References

[1] Dong E , Du H , Gardner L . An interactive web‐based dashboard to track COVID‐19 in real time. Lancet Infect Dis. 2020;20(5):533‐534. 32087114 10.1016/S1473-3099(20)30120-1 PMC7159018

[2] World Health Organization . COVID‐19 Vaccines. World Health Organization; 2022. Accessed November 18, 2022. https://www.who.int/emergencies/diseases/novel-coronavirus-2019/covid-19-vaccines

[3] Han X , Xu P , Ye Q . Analysis of COVID‐19 vaccines: types, thoughts, and application. J Clin Lab Anal. 2021;35(9):e23937. 34396586 10.1002/jcla.23937 PMC8418485

[4] World Health Organization . COVAX. World Health Organization; 2022. Accessed November 18, 2022. https://www.who.int/initiatives/act-accelerator/covax

[5] National Command Operation Center [Internet]. 2022. [cited 2022 Jan 30]. Available from: https://ncoc.gov.pk/covid-vaccination-en.php

[6] Rozek LS , Jones P , Menon A , Hicken A , Apsley S , King EJ . Understanding vaccine hesitancy in the context of COVID‐19: the role of trust and confidence in a seventeen‐country survey. Int J Public Health. 2021;66:636255. 34744589 10.3389/ijph.2021.636255 PMC8565283

[7] Nehal KR , Steendam LM , Campos Ponce M , van der Hoeven M , Smit GSA . Worldwide vaccination willingness for covid‐19: a systematic review and meta‐analysis. Vaccines. 2021;9(10):1071. 34696179 10.3390/vaccines9101071 PMC8540052

[8] Lazarus JV , Ratzan SC , Palayew A , et al. A global survey of potential acceptance of a COVID‐19 vaccine. Nature Med. 2021;27(2):225‐228. 33082575 10.1038/s41591-020-1124-9 PMC7573523

[9] Neumann‐Böhme S , Varghese NE , Sabat I , et al. Once we have it, will we use it? A European survey on willingness to be vaccinated against COVID‐19. Eur J Health Econ. 2020;21(7):977‐982. 32591957 10.1007/s10198-020-01208-6 PMC7317261

[10] Yigit M , Ozkaya‐Parlakay A , Senel E . Evaluation of COVID‐19 vaccine refusal in parents. Pediatr Infect Dis J. 2021;40(4):e134‐e136. 33410650 10.1097/INF.0000000000003042

[11] Kreps S , Prasad S , Brownstein JS , et al. Factors associated with US adults’ likelihood of accepting COVID‐19 vaccination. JAMA Network Open. 2020;3(10):e2025594. 33079199 10.1001/jamanetworkopen.2020.25594 PMC7576409

[12] Yin F , Wu Z , Xia X , Ji M , Wang Y , Hu Z . Unfolding the determinants of COVID‐19 vaccine acceptance in China. J Med Internet Res. 2021;23(1):e26089. https://pubmed.ncbi.nlm.nih.gov/33400682/ 33400682 10.2196/26089 PMC7813210

[13] Yoda T , Suksatit B , Tokuda M , Katsuyama H . Analysis of people's attitude toward COVID‐19 vaccine and its information sources in Thailand. Cureus. 2022;14(2):e22215. 35308704 10.7759/cureus.22215 PMC8926488

[14] Mubarak A , Baabbad A , Almalki N , Alrbaiai G , Alsufyani G , Kabrah D . Beliefs, barriers, and acceptance associated with COVID‐19 vaccination among Taif University students in Saudi Arabia. J Family Med Prim Care. 2022;11(1):224. 35309633 10.4103/jfmpc.jfmpc_1255_21 PMC8930104

[15] Di Giuseppe G , Pelullo CP , Volgare AS , Napolitano F , Pavia M . Parents’ willingness to vaccinate their children with COVID‐19 vaccine: results of a survey in Italy. J Adolesc Health. 2022;70(4):550‐558. 35305792 10.1016/j.jadohealth.2022.01.003 PMC8767903

[16] Moscardino U , Musso P , Inguglia C , Ceccon C , Miconi D , Rousseau C . Sociodemographic and psychological correlates of COVID‐19 vaccine hesitancy and resistance in the young adult population in Italy. Vaccine. 2022;40(16):2379‐2387. 35305828 10.1016/j.vaccine.2022.03.018 PMC8920409

[17] Mallapaty S , Ledford H . COVID‐vaccine results are on the way—and scientists’ concerns are growing. Nature. 2020;586(7827):16‐17. 32978611 10.1038/d41586-020-02706-6

[18] World Health Organization . Ten Threats to Global Health in 2019. World Health Organization; 2022. Accessed November 18, 2022. https://www.who.int/news-room/spotlight/ten-threats-to-global-health-in-2019

[19] Khan YH , Mallhi TH , Alotaibi NH , et al. Threat of COVID‐19 vaccine hesitancy in Pakistan: the need for measures to neutralize misleading narratives. Am J Trop Med Hyg. 2020;103(2):603‐604. 32588810 10.4269/ajtmh.20-0654 PMC7410483

[20] Von Elm E , Altman DG , Egger M , Pocock SJ , Gøtzsche PC , Vandenbroucke JP . The strengthening the reporting of observational studies in epidemiology (STROBE) statement: guidelines for reporting observational studies. Ann Intern Med. 2007;147(8):573‐577. 17938396 10.7326/0003-4819-147-8-200710160-00010

[21] Riad A , Pokorná A , Attia S , Klugarová J , Koščík M , Klugar M . Prevalence of covid‐19 vaccine side effects among healthcare workers in the Czech Republic. J Clin Med. 2021;10(7):1428. 33916020 10.3390/jcm10071428 PMC8037149

[22] Nittner‐Marszalska M , Rosiek‐Biegus M , Kopeć A , et al. Pfizer‐BioNTech COVID‐19 vaccine tolerance in allergic versus non‐allergic individuals. Vaccines. 2021;9(6):553. 34070671 10.3390/vaccines9060553 PMC8230004

[23] Hatmal MM , Al‐Hatamleh MAI , Olaimat AN , et al. Side effects and perceptions following covid‐19 vaccination in Jordan: a randomized, cross‐sectional study implementing machine learning for predicting severity of side effects. Vaccines. 2021;9(6):556. 34073382 10.3390/vaccines9060556 PMC8229440

[24] Zhang Y , Zeng G , Pan H , et al. Safety, tolerability, and immunogenicity of an inactivated SARS‐CoV‐2 vaccine in healthy adults aged 18–59 years: a randomised, double‐blind, placebo‐controlled, phase 1/2 clinical trial. Lancet Infect Dis. 2021;21(2):181‐192. 33217362 10.1016/S1473-3099(20)30843-4 PMC7832443

[25] El‐Shitany NA , Harakeh S , Badr‐Eldin SM , et al. Minor to moderate side effects of Pfizer‐BioNTech COVID‐19 vaccine among Saudi residents: a retrospective cross‐sectional study. Int J Gen Med. 2021;14:1389‐1401. 33907443 10.2147/IJGM.S310497 PMC8068468

[26] Menni C , Klaser K , May A , et al. Vaccine side‐effects and SARS‐CoV‐2 infection after vaccination in users of the COVID symptom study app in the UK: a prospective observational study. Lancet Infect Dis. 2021;21(7):939‐949. 33930320 10.1016/S1473-3099(21)00224-3 PMC8078878

[27] CDC . Pfizer‐BioNTech COVID‐19 Vaccine Reactions & Adverse Events. CDC; 2022. Accessed November 18, 2022. https://www.cdc.gov/vaccines/covid-19/info-by-product/pfizer/reactogenicity.html

[28] Riad A , Sağıroğlu D , Üstün B , et al. Prevalence and risk factors of CoronaVac side effects: an independent cross‐sectional study among healthcare workers in Turkey. J Clin Med. 2021;10(12):2629. 34203769 10.3390/jcm10122629 PMC8232682

[29] Choi WS , Cheong HJ . COVID‐19 vaccination for people with comorbidities. Infect Chemother. 2021;53(1):155. 34409789 10.3947/ic.2021.0302 PMC8032917

[30] Jayadevan R , Shenoy R , TS A . Survey of symptoms following COVID‐19 vaccination in India. 2021. Accessed November 18, 2022. https://www.medrxiv.org/content/10.1101/2021.02.08.21251366v1

[31] Bae S , Lee YW , Lim SY , et al. Adverse reactions following the first dose of ChAdOx1 nCoV‐19 vaccine and BNT162b2 vaccine for healthcare workers in South Korea. J Korean Med Sci. 2021;36(17):1‐9 10.3346/jkms.2021.36.e115 PMC8093607 33942579

[32] Saeed BQ , Al‐Shahrabi R , Alhaj SS , Alkokhardi ZM , Adrees AO . Side effects and perceptions following Sinopharm COVID‐19 vaccination. Int J Infect Dis. 2021;111:219‐226. 34384899 10.1016/j.ijid.2021.08.013 PMC8351310

[33] Hervé C , Laupèze B , Del Giudice G , Didierlaurent AM , Da Silva FT . The how's and what's of vaccine reactogenicity. NPJ Vaccines. 2019;4(1):1‐11. 31583123 10.1038/s41541-019-0132-6 PMC6760227

